# Is Quality of Life a Reason Related to the Retirement of Professional Male Soccer Players?

**DOI:** 10.1155/tsm2/3376033

**Published:** 2025-12-11

**Authors:** Masar Gjaka, Kaltrina Feka, Abbey Thomas, Harald Tschan, Antonio Tessitore

**Affiliations:** ^1^ Department of Sport and Movement Science, University for Business and Technology, Pristina, Kosovo, ubt-uni.net; ^2^ Department of Applied Physiology, Health, and Clinical Sciences, Visiting Fulbright Scholar at University of North Carolina at Charlotte, Charlotte, North Carolina, USA; ^3^ Department of Health Sciences, Universum College, Pristina, Kosovo; ^4^ Department of Applied Physiology, Health, and Clinical Sciences, University of North Carolina at Charlotte, Charlotte, North Carolina, USA, uncc.edu; ^5^ Department of Sport and Human Movement Science, University of Vienna, Vienna, Austria, univie.ac.at; ^6^ Department of Movement, Human and Health Sciences, University of Rome “Foro Italico”, Rome, Italy, uniroma4.it

**Keywords:** health status, professional soccer, quality of life, retired players, sport

## Abstract

**Introduction:**

Limited knowledge exists regarding how retirement from a professional soccer career may influence players’ future quality of life and overall health status. This study aimed to explore the reasons for ending professional soccer careers and to examine the postretirement health and quality of life among former soccer players in Kosovo.

**Materials and Methods:**

Seventy‐three retired male professional soccer players from Kosovo participated in this cross‐sectional study with a retrospective design. The participants completed a questionnaire covering demographics, career details, reasons for retiring, history of soccer‐related injuries, current health and activity status, and psychological aspects associated with their professional careers and retirement. Players were divided into two groups: medical retirees (MRs) and nonmedical retirees (NMRs). The Mann–Whitney *U* test was used to compare continuous data between the two groups, and cross‐tabulation methods were employed for categorical data.

**Results:**

Nonmedical reasons were the main cause of retirement among Kosovar soccer players (*p* < 0.001), with job‐related issues (58.8%) as the main retirement reason. The NMR group retired later and had longer careers than the MR group, though this was not statistically significant. Lower extremity injuries, particularly ankle and knee injuries, were the most prevalent among all players. After retirement, the NMR group engaged more in physical activity and continued playing soccer significantly more than the MR group (*p* < 0.001 and *p* = 0.021). The MR group reported significantly more pain (*p* < 0.001), analgesic use (*p* = 0.001), fear of career‐ending injuries (*p* < 0.001), and depression symptoms during their careers (*p* < 0.001). Overall, NMR had significantly better quality of life and health status than MR (*p* < 0.001 and *p* < 0.001, respectively).

**Conclusions:**

This study highlights the impact of retirement reasons on the long‐term health and quality of life of soccer players in Kosovo. It calls for enhanced support systems to prepare athletes for life after soccer, mitigating negative health outcomes associated with forced retirement due to injuries.

## 1. Introduction

The delicate balance between physical and psychosocial load and recovery is a constant challenge for players in elite soccer, as well as in other elite team sports, in order to maintain their performance at a high level and prevent injuries and illnesses [1, 2]. Soccer‐related injuries not only negatively impact the performance of their own team [3], but they also signify the uncertain nature of their professional careers as well as their quality of life [3]. This uncertainty can lead to long‐term health consequences for players, such as an increased risk of neurodegenerative diseases [4], musculoskeletal pain [5, 6], and poor mental health symptoms [7]. In light of the aforementioned information, rehabilitation strategies that are designed to restore players’ abilities in the shortest possible time have recently received growing attention [8]. In this regard, research groups are currently advocating for injury prevention strategies to enable athletes to extend their professional careers [9].

There is a general agreement that elite athletes have better long‐term health status compared to the general population [10, 11]. Nevertheless, retirement from professional sports leads to reduced physical activity levels, which may increase body mass index (BMI) [12]. This combination of reduced physical activity and higher BMI may increase cardiovascular disease risk [13]. Additionally, studies have demonstrated that former soccer players compared to in‐career ones are more likely to get involved in unhealthy behaviors such as smoking, alcohol, and poor nutrition [14, 15] that may further impair physical activity, BMI, and cardiovascular disease risk.

Discontinuation reasons and their relationship with the health‐related quality of life (HRQoL) have been investigated among former soccer players [9], who reported a lower quality of life among former players who discontinued their career due to injuries. To highlight the importance of this aspect, a study by Kuenze et al. [16] involving former American football players (NFL level) showed that lower extremity joint injuries are even associated with higher risks of cardiovascular disease. Additionally, DeFreese et al. [17] reported a significant correlation between depression and anxiety and involuntary discontinuation among players without a transition plan.

Several health conditions encountered by retired professional soccer players have been identified, which may help pinpoint appropriate proactive strategies during and after a player’s career in order to mitigate poor long‐term health risks [18]. Consequently, studies have called for the establishment of specific interventions that may help retired soccer players. Indeed, researchers have initiated initiatives such as concussion management, musculoskeletal injury prevention programs, load monitoring, and improving understanding of treatment options for soccer‐related injuries during the players’ active careers [19]. However, to the best of the authors’ knowledge, there is currently a lack of information regarding the reasons behind the retirement of professional soccer players in the Republic of Kosovo, as well as their health status.

Therefore, the aim of this study was to conduct a retrospective analysis to identify the key factors contributing to the retirement of professional Kosovar male soccer players from health, psychological, and social perspectives. We hypothesize that the causes of career cessation differ between players with medical and nonmedical reasons for retirement, with nonmedical retirees (NMRs) expected to report higher postretirement health status and quality of life.

## 2. Materials and Methods

### 2.1. Participants

An online, self‐administered survey was sent to a total of 185 male retired soccer players, with 86 responses (response rate: 46.5%) received from players who voluntarily participated in the study. However, since 13 surveys were incomplete, a final total of 73 surveys (response rate: 39.5%) were included in the statistical analysis. Table 1 presents the general characteristics of the participants, all of whom provided written informed consent before completing the survey.

**Table 1:** Anthropometric and football‐specific data of retired professional soccer players.

		Total *n* = 73	Medical retirees, *n* = 22	Non‐medical retirees, *n* = 51
Age at the end of professional career	Mean ± SD (95% CI) (y)	29.03 ± 5.05 (27.85–30.21)	28.50 ± 5.4 (26.10–30.90)	29.25 ± 4.93 (27.87–30.64)
Duration of complete football career	Mean ± SD (95% CI) (y)	16.16 ± 3.95 (15.24–17.09)	16 ± 4.27 (14.10–17.90)	16.24 ± 3.85 (15.15–17.32)
Years between career end and the survey	Mean ± SD (95% CI) (y)	10.52 ± 6.67 (8.96–12.07)	8.63 ± 5.86 (6.03–11.23)	11.33 ± 6.89 (9.39–13.27)
Official matches				
1–50	*n* (%)	3 (4.1)	1 (4.5)	2 (3.9)
51–100		9 (12.3)	2 (9.1)	7 (13.7)
101–200		19 (26.0)	7 (31.8)	12 (23.5)
201–300		14 (19.2)	6 (27.3)	8 (15.7)
> 300		28 (38.4)	6 (27.3)	22 (43.1)
Dominant leg				
Right	*n* (%)	60 (82.2)	17 (77.3)	43 (84.3)
Left		13 (17.8)	5 (22.7)	8 (15.7)
Playing position				
Goalkeeper	*n* (%)	3 (4.1)	1 (4.5)	2 (3.9)
Defender		25 (34.2)	9 (40.9)	16 (31.4)
Midfielder		26 (35.6)	7 (31.8)	19 (37.3)
Striker		19 (26)	5 (22.7)	14 (27.5)
Body mass index				
During career	Mean ± SD (95% CI)	25.69 ± 1.53 (25.33–26.05)	25.95 ± 1.63 (25.23–26.68)	25.58 ± 1.49 (25.16–26)
After career		29.36 ± 3.21 (28.61–30.11)^∗^	28.97 ± 3.73 (27.32–30.63)	29.53 ± 2.98 (28.69–30.37)
Level of education				
Middle school	*n* (%)	12 (16.4)	5 (22.7)	7 (13.7)
High school		5 (6.8)	1 (4.5)	4 (7.8)
Vocational school		3 (4.1)	2 (9.1)	1 (2)
Bachelor		28 (38.4)	7 (31.8)	21 (41.2)
Master		24 (32.9)	6 (27.3)	18 (35.3)
PhD		1 (1.4)	1 (4.5)	—

^∗^Significantly higher compared to during professional career (*p* < 0.05).

### 2.2. Procedures

The local ethical committee of the University for Business and Technology (Prot. Nr. 12898/45, date: 25.08.2022) approved the design and methods of this study. A cross‐sectional study incorporating a retrospective design for some questions was used to survey and analyze the reasons for the end of professional careers and the current health status of retired professional male Kosovar soccer players. Through the Football Federation of Kosovo (Federata e Futbollit e Kosovës), an ad hoc survey along with study guidelines was distributed to retired players. Additionally, the list of participants was further expanded through contacts with soccer clubs and personal direct contacts. The survey was open for completion from September 1, 2022, to February 28, 2023. Participants were exclusively former professional soccer players who had at least 3 years of retirement at the time of survey distribution. Professional players were defined as those who had a professional contract and had played with a team in the Professional Football League or the First League, which represent the two highest levels of soccer competition in the Republic of Kosovo. The survey included items related to participants’ demographics, career details, reasons for ending their careers, history of soccer‐related injuries, current health and activity status, and psychological aspects associated with their professional soccer careers and retirement (see Supporting Information). Most of the questions used in the survey were binary (e.g., yes or no) and were related to the periods during and after retirement. The ad hoc survey was developed based on standardized injury surveys previously used by Hägglund et al. (2005) and Fuller et al. [20]. Additionally, this study adopted the approach outlined by Koch et al. [9]. Specifically, participants were divided into two groups based on their reasons for ending their professional careers. The first group (medical retirees [MR]) consisted of players who reported retiring due to one or more injuries. The second group NMR included players who did not report medical reasons for their retirement.

### 2.3. Statistical Analysis

Statistical analysis was conducted using SPSS^®^ (Version 25, IBM, Armonk, NY, USA). Since no specific primary endpoint existed, no sample size calculation was performed beforehand. The Kolmogorov–Smirnov test was used to test the assumption of normality of data. The data are presented as mean ± standard deviation (SD) and 95% confidence interval (CI) for continuous data and as absolute and relative frequencies for categorical data. The Mann–Whitney *U* test was used to compare continuous data between the two groups. For categorical data, cross‐tabulation methods with the *χ* ^2^ test were employed. A probability of *p* ≤ 0.05 was considered significant.

## 3. Results

A greater number of respondents retired due to nonmedical reasons (*n* = 51; 60.9%) compared to medical ones (*n* = 22; 30.1%) (*p*  <  0.001). Overall, there was a notable rise in mean BMI (*p*  <  0.001) (Table 1) after retiring from professional soccer. Although not statistically significant, results demonstrated that players belonging to NMR retired at an older age and had a longer career compared to MR (Table 1). Regarding the nonmedical reasons for retirement, alternative job‐related aspects were the most prevalent, followed by personal reasons and, ultimately, age (Table 2).

**Table 2:** Reasons for the retirement from a professional soccer career.

Reasons for retirement		Medical retirees, *n* = 22 *n* (%)	Non‐medical retirees, *n* = 51 *n* (%)
Medical	Acute injury	13 (59.1)	—
	Chronic injury/injuries	9 (40.9)	—
	Total	22 (100)	—
Nonmedical	Age	—	9 (17.6)
	Alternative job	—	30 (58.8)
	Personal reasons	—	12 (23.5)
	Total	—	51 (100)

In total, 187 injuries were reported, with the ankle (22.5%) and the knee (20.9%) being the most affected body regions (Figure 1(a)). Sprain/ligament injuries (*n* = 49), bruise (*n* = 31), and dislocation/subluxation (*n* = 28) followed by fracture (*n* = 24) and muscle rupture/strain/tear injuries (*n* = 22) (Figure 1(b)) were the most prevalent.

Figure 1Frequency of injury by (a) body regions and (b) type of injury.

**Figure:**
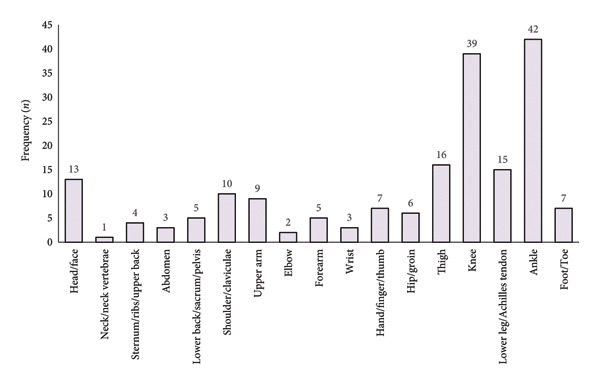
(a)

**Figure:**
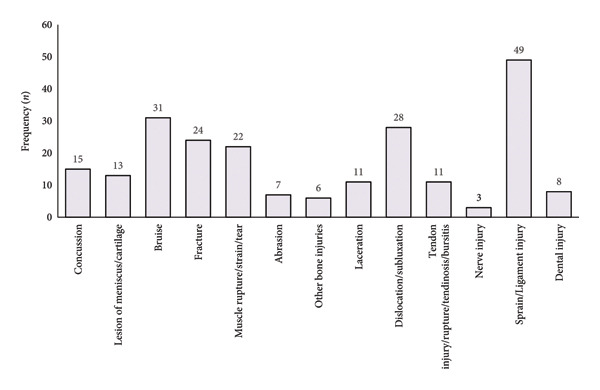
(b)

Nearly all respondents (98%) in the NMR group reported current involvement in physical activity compared to 68.2% in the MR group (*p*  <  0.001) (Table 3). Regarding the continuation of soccer after retirement, 59.1% of the MR group compared with 84.3% of the NMR group still played recreational soccer (*p* = 0.021). Furthermore, twice as many respondents in the MR group experienced symptoms such as pain (*p* = 0.001) and over five times more used analgesics (*p*  <  0.001) compared with the NMR group. Finally, 19 respondents were diagnosed with osteoarthritis, with no significant differences observed between groups (Table 3).

**Table 3:** Activity and health status after retirement from a professional soccer career.

	**Total (*n* = 73)**	**Medical retirees, *n* = 22**	**Non-medical retirees, *n* = 51**
***n* (%)**			

PA involvement			
Yes	65 (89)	15 (68.2)	50 (98)^∗∗^
No	8 (11)	7 (31.8)	1 (2)
Playing football			
Yes	56 (76.7)	13 (59.1)	43 (84.3)^∗^
No	15 (20.5)	8 (36.4)	7 (13.7)
Assistive equipment	2 (2.7)	1 (4.5)	1 (2)
Symptoms			
Pain	21 (28.8)	14 (63.6)^∗^	7 (13.7)
Instability	15 (20.5)	8 (36.4)	7 (13.7)
Effusion	4 (5.5)	3 (13.6)	1 (2)
Diagnosed osteoarthritis			
Hip	4 (5.5)	2 (9.1)	2 (3.9)
Knee	7 (9.6)	4 (18.2)	3 (5.9)
Ankle	8 (11)	4 (18.2)	4 (7.8)
Using analgesics			
Yes	20 (27.4)	17 (77.3)^∗∗^	3 (5.9)
No	53 (72.6)	5 (22.7)	48 (94.1)

^∗∗^Significant differences at *p* < 0.001.

^∗^Significant differences at *p* < 0.05.

Players in the MR group (72.7%) showed a significantly higher rate of depression symptoms during their career compared to those in the NMR group (5.9%) (*p*  <  0.001) (Table 4). Following retirement, there was a noticeable reduction in overall depression rates; however, the MR group still reported significantly elevated levels (*p* = 0.009) compared to the NMR group. Moreover, more respondents in the MR group reported that during their career, they feared that they would sustain a career‐ending injury (*p*  <  0.001). Conversely, there were no differences in negative emotions upon retiring from professional football. However, players in the NMR group compared to the MR group were much more likely to have postsoccer career plans (*p*  <  0.001). Only a small number of retired players faced challenges transitioning from soccer to their current jobs, and the reason for retirement had no significant effect. As expected, retired players in the NMR group reported significantly better health outcomes (*p*  <  0.001) and a higher quality of life (*p* = 0.001) after retirement (Table 4).

**Table 4:** Psychological aspects related to a professional soccer career and retirement.

		Total (*n* = 73)	Medical retirees, *n* = 22	Non‐medical retirees, *n* = 51
Depression				
During career	*n* (%)	19 (26)	16 (72.7)^∗∗^	3 (5.9)
After retirement		11 (15.1)	7 (31.8)^∗^	4 (7.8)
Fear of career‐end injury	*n* (%)	29 (39.7)	18 (81.8)^∗∗^	11 (21.6)
Negative emotions at the end of career	*n* (%)	43 (58.9)	18 (81.8)	25 (49)
Prepared for future career	*n* (%)	49 (67.1)	8 (36.4)	41 (80.4)^∗∗^
Transition problems from professional career to current employment	*n* (%)	16 (21.9)	7 (31.8)	9 (17.6)
Football‐related employment	*n* (%)	45 (61.6)	17 (77.3)	28 (54.9)
Current health condition	Mean ± SD (95% CI)	2.60 ± 0.77 (2.42–2.78)	3.36 ± 0.49 (3.15–3.58)	2.27 ± 0.63 (2.79–3.3)^∗∗^
Current quality of life		2.62 ± 0.71 (2.45–2.78)	3.05 ± 0.57 (2.10–2.45)	2.43 ± 0.70 (2.23–2.63)^∗^

^∗∗^Significant differences at *p* < 0.001.

^∗^Significant differences at *p* < 0.05.

## 4. Discussion

This retrospective study aimed to investigate the factors and health status at the time of professional career cessation, as well as the quality of life after retirement, among Kosovar professional soccer players. The study demonstrated, for the first time, that nonmedical factors were the primary cause of termination of professional soccer careers in the Republic of Kosovo (*p*  <  0.001). Alternative job‐related features were the most common cause for the end of a career, followed by personal reasons and finally age. The findings of our study disagree with those of Koch et al. [9], where medical reasons (e.g., acute and chronic injuries) were reported as the main reason for ending a professional soccer career in the Bundesliga and Bundesliga 1, which are the highest soccer leagues in Germany. Significantly lower annual revenue, along with political, social, and other factors, could account for this discrepancy, potentially explaining why Kosovar professional soccer players might find other careers more financially rewarding than soccer.

Furthermore, participants in the NMR group reported higher ratings for their current health status and quality of life compared to those in the MR group. These findings align with existing research suggesting an association between lower quality of life or life satisfaction and involuntary retirement from sports when exploring the impact of retirement on health status [21].

Regarding the number of injuries among male professional soccer players, Ekstrand et al. [22] reported a decrease in the rate over the last two decades. Nevertheless, irrespective of the reason for a career‐ending injury, the findings of the present study indicate that the lower extremities (e.g., knee and ankle) are the most commonly affected body regions in soccer‐related injuries. Furthermore, the findings of the current research on injury distribution are in complete agreement with the related literature [3, 23]. Indeed, there is widespread recognition of the degenerative nature and high frequency of injuries in professional soccer [24, 25]. This recognition extends to the prevalence of injuries among professional soccer players in the Republic of Kosovo, as well as the potential prognostic factors for specific soccer‐related injuries, like hamstring injuries [26, 27].

The presence of symptoms such as pain or joint instability is associated with the onset of osteoarthritis, especially in the knee and ankle joints. In fact, Drawer and Fuller [28] have previously demonstrated an increased incidence of injuries per joint and an elevated risk of developing osteoarthritis over two decades. The present study consistently observed higher pain levels in the MR group compared to the NMR group. Although there were no statistically significant differences between the two groups, the body locations most commonly affected by osteoarthritis were the ankle and knee. These findings partially align with the literature, which reports that osteoarthritis primarily affects the knee joint, with the ankle and hip following [9]. The higher number of reported pain cases in the MR group is likely related to the significantly higher number of retired players who disclosed the use of analgesics, though the statistical analysis performed does not allow for cause/effect determination.

Regarding physical activity involvement after retirement, the decision to retire due to medical reasons had an impact on both physical activity participation and playing soccer. Specifically, the majority of the retired professional soccer players reported being physically active, though the NMR group was significantly more active than the MR one. Additionally, the NMR group showed significantly higher involvement in playing soccer compared to players who ended their professional soccer careers due to medical reasons. The results reported by Koch et al. [9] support these findings. Ultimately, the Football Veterans’ League, a rare organization even among more developed countries, is largely responsible for the involvement of retired players in physical activity, particularly in soccer.

This study also aimed to evaluate the impact of retirement reasons (e.g., medical or nonmedical) on the overall health status of retired professional soccer players. In addition to examining their current health status, the analysis also examined their psychological well‐being postretirement and their present quality of life. Regardless of their group, 26% of retired players in our study reported experiencing depression during their careers. The present results are in contrast with earlier studies, which reported higher percentage rates of depression cases ranging from 35% to 39% [7, 14]. Notably, there were significant disparities observed between the two groups, with a higher number of cases reported among the MR group. The prevalence of depression among players after retiring from professional soccer leveled off but still showed significant differences between the two groups, which is in line with the existing literature [29]. Given that episodes of depression were found to be significantly more prevalent among players who had retired due to injury, it can be inferred that there is an association between psychological health issues (depression and anxiety) and the occurrence of injuries [14, 30]. Hence, reducing injury risk may reduce the prevalence of depression among professional soccer players. Alternatively, when injuries are not able to be prevented, it appears crucial to prioritize the resolution of mental health problems concurrent with physical injury healing and raise awareness about psychological well‐being among active players [9].

This study demonstrated that significantly higher numbers of players who retired due to medical reasons were already concerned about the possibility of sustaining career‐ending injuries while still actively playing professional soccer. Therefore, it could be speculated that anxious players may find it more challenging to cope with the reality of premature retirement due to medical reasons compared to nonanxious players. This could be managed better if the professional players were prepared for the transition from a professional career to the job market. In fact, the present results showed that players belonging to the NMR group were more prepared for a career after retiring from professional soccer compared to the MR group. In this respect, relevant stakeholders in Kosovo should make substantial efforts to foster professional athletes in general and soccer players in particular to pursue their dual careers [31].

Despite the strengths and uniqueness of the present study, there are limitations that should be acknowledged when interpreting the results. Despite our collaboration with the Football Federation of Kosovo and the Veterans’ League, the overall sample size of this study is limited. Further, the survey employed in the study used retrospective questions which might be subject to the recall bias of the respondents. However, the retrospective nature of the questions used offered the participants the opportunity to reflect on their experiences in ways that could not have been accessed by other approaches.

## 5. Conclusion

The present study’s results offer valuable insights into the key factors that contributed to the longevity of professional soccer careers in Kosovo. Specifically, players forced to retire due to medical reasons (MR group) tended to rate their health status and quality of life after retirement significantly worse than those who voluntarily ended their careers (NMR group). The results of this study should encourage the organization of awareness campaigns in order to better prepare the active professional soccer players for their postprofessional careers.

## Conflicts of Interest

The authors declare no conflicts of interest.

## Funding

The authors received no specific funding for this work.

## Supporting Information

The complete survey which was used to collect the data.

## Supporting information


**Supporting Information** Additional supporting information can be found online in the Supporting Information section.

## Data Availability

The data that support the findings of this study are available on request from the corresponding author. The data are not publicly available due to privacy or ethical restrictions.
