# Supplementary material for: Is Quality of Life a Reason Related to the Retirement of Professional Male Soccer Players?
Source: Transl Sports Med. 2025 Dec 11;2025:3376033. doi: 10.1155/tsm2/3376033 (PMC12782323; doi:10.1155/tsm2/3376033)
Supplement: Supplementary file 1 — Supporting Information Additional supporting information can be found online in the Supporting Information section. [file TSM2-2025-3376033-s001.docx]

Supplementary material 1 - SURVEYTop of Form

Demographic and football-specific data among former professional soccer players.

**What is your birthday? (dd/mm/yy)**

__________

**At what age did you finish your career? (e.g. 35)**

__________

**How long was your soccer career? (write number of years)**

__________

**In which level have you played?**

- Super League
- First League
- Both levels

**Approximately how many games did you play during your professional career?**

- 1-50
- 51-100
- 101-200
- 201-300
- >300

**Which is your dominant leg?**

- Right
- Left

**What position did you play primarily?**

- Goalkeeper
- Defender
- Midfielder
- Forward

**What is your body height? (m)**

_________

**What was your body mass during your professional football career? (kg)**

_________

**What is your current body mass/weight? (kg)**

_________

**What is the highest level of education you completed?**

- Middle school
- High school
- Vocational school
- Bachelor's degree
- Master's degree
- PhD,

**What were the reasons for your career end?**

- Medical
- Non-medical

Non-medical reasons for career end

- Age
- Alternative job
- Personal reasons

Medical reasons for career end

- Acute injury
- Chronic injury/ series of injuries

Injury occurrence and injured body parts of former professional soccer players during their career

**Did you have any significant injuries during your professional soccer career?** *(Select all body parts that apply)*

- head/face
- neck/cervical spine
- sternum/ribs/upper back
- abdomen
- lower back/pelvis/sacrum
- shoulder/clavicula
- arm
- elbow
- forearm
- wrist
- hand/finger/thumb
- hip
- thigh
- knee
- lower leg/Achilles tendon
- ankle
- foot/toe

**What type of injury did you suffer?** *(Select all types of injury that apply)*

- Fracture
- Other bone injuries
- Dislocation/subluxation
- Sprain/ligament injury
- Lesion of meniscus or cartilage
- Muscle rupture/tear/strain/cramps
- Tendon injury/rupture/tendinosis/bursitis
- Haematoma/contusion/bruise
- Laceration and skin lesion
- Abrasion
- Laceration
- Concussion (with or without loss of consciousness)
- Nerve injury
- Other Dental injuries
- Other injuries______

Activity and health status after ending professional career as a footballer

**Are you involved in any kind of physical activity?**

- Yes
- No

**Do you play recreational soccer?**

- Yes
- No

**Do you have any of the following symptoms?**

- Pain
- Instability
- Effusion
- None

**Are you diagnosed with osteoarthritis in any of the following body locations?**

- Hip
- Knee
- Ankle
- Shoulder
- Other:______
- None

**Do you use analgesics?**

- Yes
- No

Psychological aspects related to soccer career and retirement

*Please respond with Yes or No in the following questions.*

**Did you have Yes No**

Depression during career?

Post-retirement depression?

Fear of a career-ending injury during your soccer career

Negative emotions at the end of your career

Preparation for the future professional career at the end of the career

Problems in the transition from professional career to current job

Employment in the football sector after the end of the professional career

Subjective evaluation of current health status and quality of life

*The subjective evaluation of the current status is made on a scale of 1 (Excellent), 2 (Very Good), 3 (Good), 4 (Reduced), 5 (Poor)*

**How do you evaluate your current health condition?**

- 1 - Excellent
- 2 - Very good
- 3 - Good
- 4 – Reduced
- 5 – Poor

**How do you evaluate your current quality of life?**

- 1 - Excellent
- 2 - Very good
- 3 - Good
- 4 – Reduced
- 5 – Poor
